# The “Transdural” Paradox: The Case of a Paraclinoid Internal Carotid Artery Aneurysm With an Intradural Neck and Extradural Dome

**DOI:** 10.7759/cureus.115301

**Published:** 2026-08-27

**Authors:** Jihad Abdelgadir, Jeffrey Nadel, Vance Mortimer, William Couldwell

**Affiliations:** 1 Neurosurgery, University of Utah, Salt Lake City, USA

**Keywords:** aneurysm, carotid cave, clinoidectomy, distal dural ring, intradural, paraclinoid aneurysm, transdural

## Abstract

Paraclinoid internal carotid artery (ICA) aneurysms pose a surgical challenge because of their complex relationship with the distal dural ring (DDR), the boundary between the extradural cavernous sinus and the intradural subarachnoid space. The classification of these aneurysms as strictly intradural or extradural is often complicated by the anatomical variability of the DDR, which can form a dural pouch known as the carotid cave. This anatomical paradox can lead to a “transdural” aneurysm, in which the dome is in the extradural or intradural space, which poses a risk for subarachnoid hemorrhage. The unreliability of preoperative imaging and bony landmarks in definitively classifying these lesions necessitates a flexible and adaptive nomenclature and surgical approach, which is discussed herein. We describe the case of a 37-year-old man with a paraclinoid ICA aneurysm that was found to have an intradural neck and an extradural dome. We performed a combined intradural-extradural operation, which began with meticulous intradural dissection to expose the subarachnoid carotid artery for distal control, followed by an intradural anterior clinoidectomy. This case highlights the critical importance of a surgical strategy that is responsive to intraoperative anatomical findings, especially for paraclinoid aneurysms that defy simple classification.

## Introduction

Aneurysms of the paraclinoid segment of the internal carotid artery (ICA) present a challenge in vascular neurosurgery. The “paraclinoid” region, which extends from the superior point of the cavernous sinus to the ophthalmic artery origin, serves as a critical transition point where the ICA exits the extradural space and enters the intradural subarachnoid space [1-3]. The anatomical boundary between these two compartments is the distal dural ring (DDR), a structure whose integrity is crucial for dictating the risk of subarachnoid hemorrhage (SAH) [4-7]. Whereas aneurysms distal to the DDR are intradural and thus pose a risk of causing SAH, those confined to the extradural space are often more benign and do not [4,5]. This binary classification, however, is an oversimplification. A third category, termed “transdural” or “transitional,” describes aneurysms that straddle the DDR [5]. These aneurysms are a direct consequence of the intricate and asymmetrical anatomy of the DDR itself, which adheres tightly to the lateral and anterior walls of the ICA, but whose medial and posterior aspects are often redundant, folding inward to form a small dural pocket known as the carotid cave [8]. An aneurysm can arise from the intradural space within the carotid cave but then project inferiorly into the extradural space. This paradoxical anatomy is difficult to predict with conventional imaging alone, because computed tomography (CT), magnetic resonance imaging, and diagnostic angiography lack the spatial resolution to identify whether a portion of an aneurysm remains intradural [6,9]. Moreover, preoperative imaging and the use of bony landmarks as a proxy for the DDR have proven to be unreliable indicators of an aneurysm’s true location [10]. This report describes a case that depicts the anatomical paradox of a paraclinoid aneurysm with an intradural neck and extradural dome and demonstrates a specialized surgical approach to achieve successful obliteration. It also underscores the need for awareness that paraclinoid aneurysms may have intradural components, despite imaging that suggests the aneurysm is purely extradural.

## Case presentation

**Figure 1:**
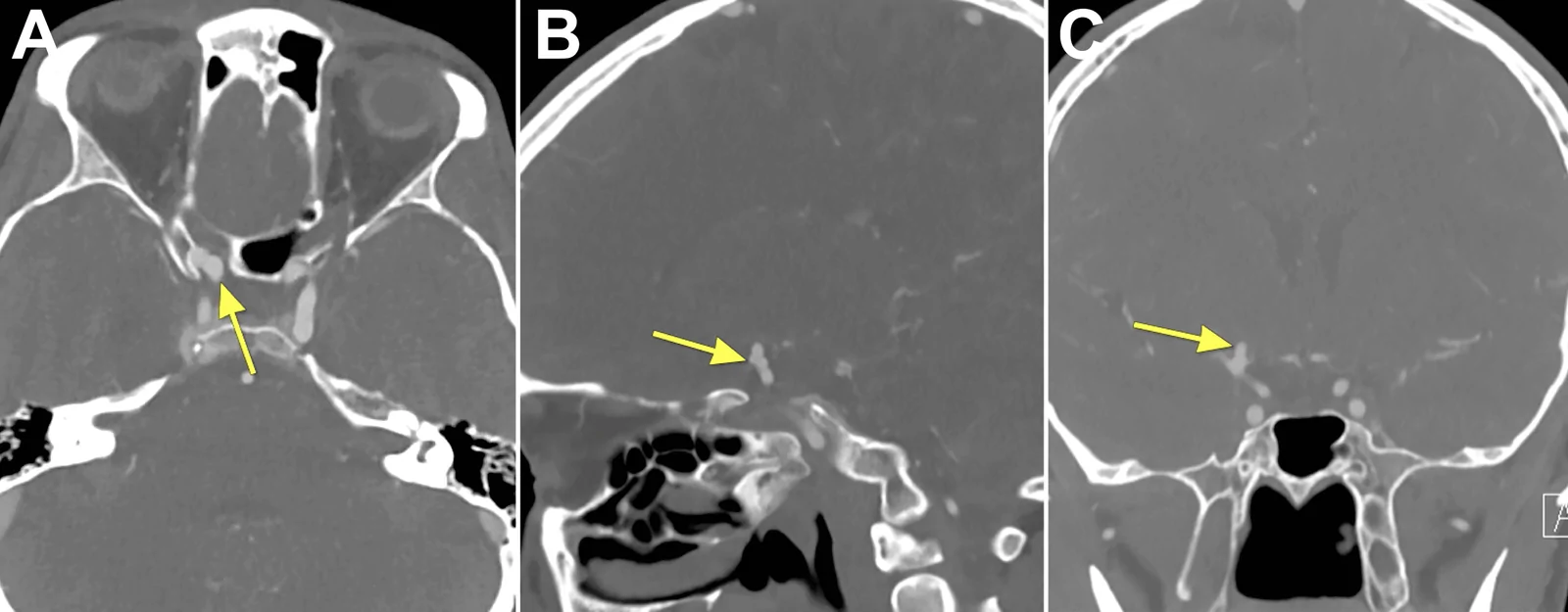
Computed tomography angiography images showing ICA terminus and paraclinoid ICA aneurysms (A) Axial, (B) sagittal, and (C) coronal images demonstrate a 4 × 3-mm anterolaterally projecting aneurysm arising from the right cavernous ICA, as well as a bilobed right carotid terminus aneurysm projecting superiorly and anteriorly, measuring 5 × 3 mm. ICA: internal carotid artery

**Figure 2:**
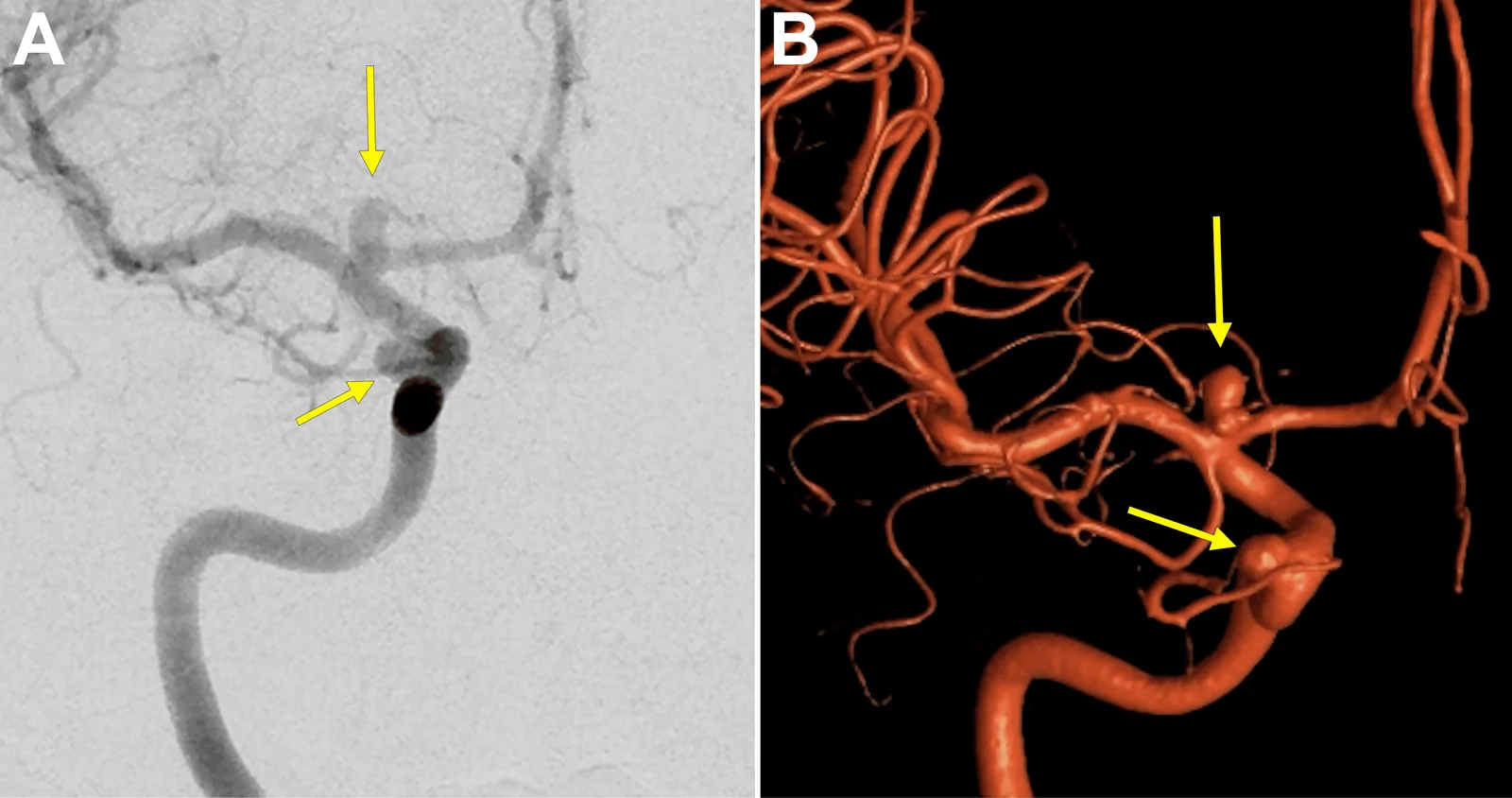
Angiography images showing ICA terminus and paraclinoid ICA aneurysms (A) Catheter angiography demonstrates a 4 × 4-mm paraclinoid aneurysm and a 5.5 × 3-mm superiorly projecting carotid terminus aneurysm with two anteriorly directed daughter sacs. (B) 3D rotational angiography shows both aneurysms. ICA: internal carotid artery

In April 2025, a 37-year-old man, an active smoker with no other relevant comorbidities, presented with a single episode of transient visual disturbance and headache lasting approximately 10 minutes, which then resolved. Given concern for a transient ischemic event, migraine with aura, or an underlying structural or vascular lesion, including the possibility of a sentinel hemorrhage from an unruptured aneurysm, lumbar puncture was performed and was negative for red blood cells and xanthochromia. Neurological examination was unremarkable, with no focal deficit. CT angiography was subsequently obtained and revealed dual intracranial vascular pathology: a 5.5-mm aneurysm at the ICA terminus and a separate 4-mm paraclinoid ICA aneurysm (Figures 1-2). He was discharged with close outpatient follow-up to discuss definitive treatment, and a right frontotemporal craniotomy for microsurgical clip reconstruction of both aneurysms was subsequently planned.

The patient was positioned supine with his head secured in a three-pin fixation device. His head was turned slightly to the left and extended, exposing the right frontotemporal region. Intraoperative neuromonitoring leads were placed to monitor motor evoked and somatosensory evoked potentials and to record electrocorticography. After a standard right-sided pterional craniotomy was performed, the surgical exposure was deepened by drilling the sphenoid wing to provide a wide, unobstructed view of the anterior skull base. Under the operative microscope (Video 1), using dynamic retraction and a subfrontal approach, we identified the olfactory nerve, the optic nerve, and then the ICA.

**Video 1:** Combined intradural-extradural microsurgical clipping of a transdural paraclinoid internal carotid artery aneurysm

**Figure 3:**
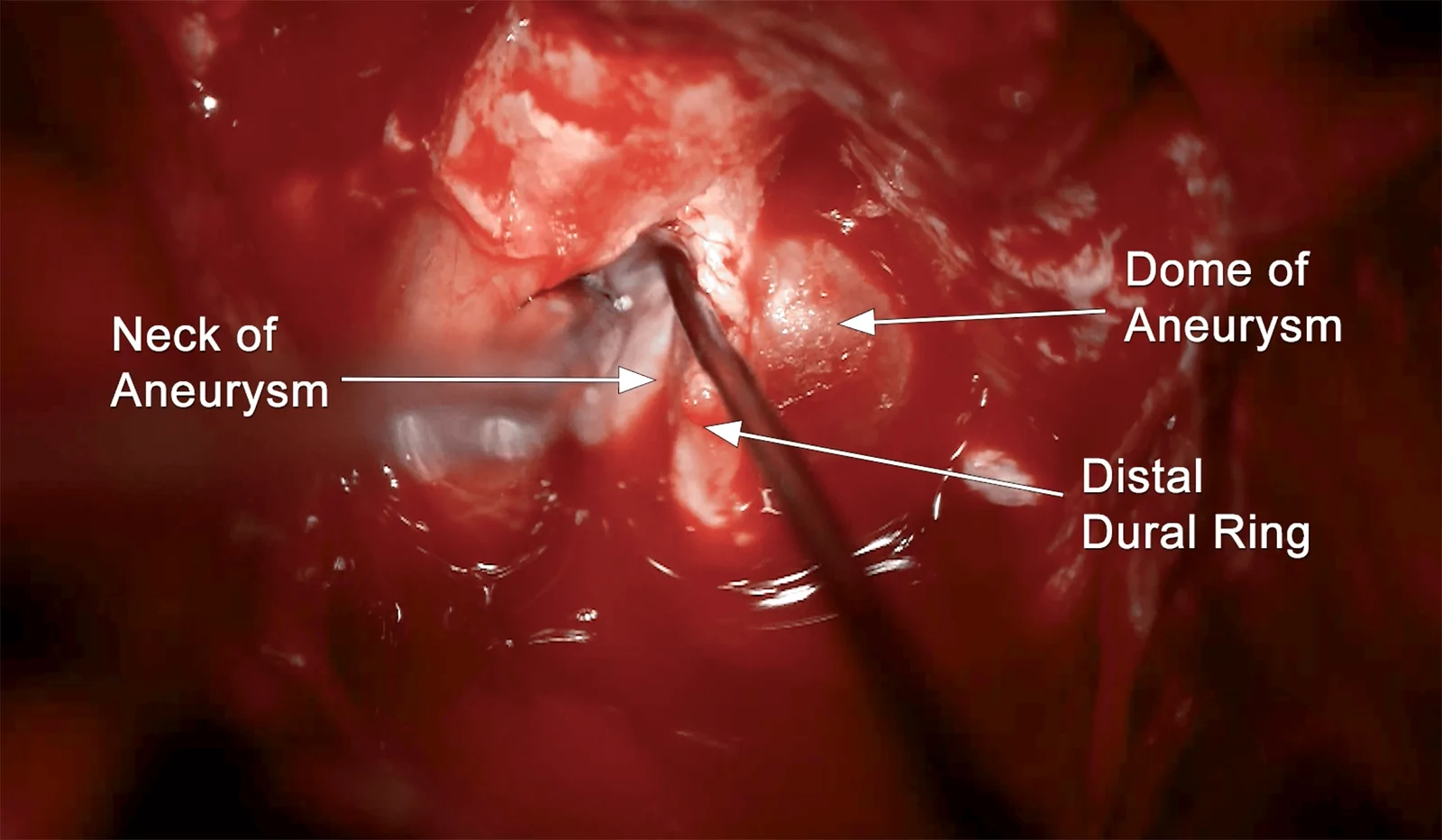
Intraoperative photograph demonstrating the paraclinoid aneurysm straddling the distal dural ring, highlighting its intradural and extradural components

The opticocarotid cistern was opened sharply to drain cerebrospinal fluid, and the Sylvian fissure was split to expose the more distal ICA terminus aneurysm first. During our evaluation of its relationship with the paraclinoid aneurysm, we discovered that the neck of the paraclinoid aneurysm was visible intradurally, distal to the DDR, whereas the dome was embedded in the anterior clinoid process (Figure 3).

To manage this complex anatomy, an intradural anterior clinoidectomy was performed. The anterior skull base dura was cut and dissected from the anterior clinoid and optic canal. The clinoid was carefully drilled using a 3-mm diamond drill to expose the DDR, the intradural aneurysm neck, and the aneurysm dome. A temporary clip was placed on the distal intradural ICA to complete the rest of the dural and bony exposure. The DDR was sharply cut, and the optic strut was carefully removed using a combination of a 1-mm diamond burr and 1-mm Kerrison rongeurs. This process fully exposed the neck of the aneurysm. The superior wall of the cavernous sinus was then opened to visualize the extradural aneurysm dome. Bleeding from the cavernous sinus was controlled with a hemostatic agent. A permanent clip was placed across the neck of the paraclinoid aneurysm, including the sharply cut DDR. Intraoperative indocyanine green videoangiography confirmed complete obliteration with patent proximal and distal blood flow.

Attention was then turned to the distal carotid terminus aneurysm. The M1 segment, the A1 segment, and the aneurysm itself were sharply dissected until circumferential exposure was attained. A temporary clip was placed on the ICA just proximal to the bifurcation to soften the aneurysm dome, and an angled permanent clip was placed across the neck of the aneurysm. The temporary clip was removed, and indocyanine green videoangiography and Doppler flow ultrasound confirmed the technical success, demonstrating complete obliteration with preservation of flow in the parent vessels. Finally, the dura was closed, the craniotomy flap was secured, and the wound was closed in layers.

Postoperative CT angiography demonstrated complete obliteration of both aneurysms without vascular compromise. The patient recovered without new neurological deficits and was discharged home on postoperative day 3. No perioperative complications occurred. Outpatient follow-up was scheduled; at the time of this report, longer-term clinical and imaging follow-up had not yet occurred.

## Discussion

This case provides a compelling illustration of the anatomical and surgical complexities inherent in managing paraclinoid ICA aneurysms, particularly those with a transdural morphology. In this patient’s case, the intradural aneurysm neck and partial extradural dome are a classic example of this paradoxical anatomy. The existence of such a lesion is anatomically plausible because of the carotid cave, a potential space formed by the redundancy, or inferior involution, of the DDR [4,5,7,8]. An aneurysm arising from this intradural pouch can expand inferiorly into the extradural cavernous sinus, appearing on imaging as an extradural lesion despite retaining an intradural neck and thus a risk of causing SAH.

Consistent with prior reports [9-12], this case illustrates that preoperative imaging and bony landmarks can be unreliable for definitive classification. A study of 15 patients, all with paraclinoid aneurysms that caused SAH and were thus confirmed to be intradural, demonstrated the limitations of using bony landmarks alone to stratify patient risk [10]. This significant rate of misclassification highlights the need for a flexible management strategy.

Our findings are consistent with, and extend, prior reports describing this paradoxical anatomy. Ribeiro et al. [13] described a paraclinoid aneurysm with an intradural neck and an extradural, intrasellar dome that ruptured, causing subarachnoid and intrasellar hemorrhage despite an ambiguous appearance on CT and CT angiography; magnetic resonance imaging was required to confirm the intradural component, and this finding guided the team toward endovascular coiling. Our patient's aneurysm shared this same intradural neck, extradural dome configuration but presented unruptured, was identified alongside a second, concurrent carotid terminus aneurysm, and was treated surgically rather than endovascularly. This contrast illustrates that the same anatomical variant can span a spectrum of clinical severity, from incidental discovery to SAH, and that treatment selection depends on more than the transdural anatomy alone.

de Morais et al. [14] similarly reported two carotid cave aneurysms treated via an extradural-first anterior clinoidectomy, reasoning that this sequence minimizes operative time and avoids contaminating the subarachnoid space with bone dust. Our operative sequence differed: because the paraclinoid aneurysm's neck was intradural and because concurrent intradural exposure was already required for proximal control of the second, unrelated carotid terminus aneurysm, we elected an intradural-first strategy. This difference, despite similar carotid cave anatomy, illustrates that no single fixed sequence of intradural versus extradural exposure is universally superior; rather, the operative sequence should be individualized to the aneurysm's specific configuration and any concurrent pathology.

Given the potential for preoperative uncertainty, the surgical methodology in this case was dictated by a logical, multimodal strategy with a flexible and adaptive approach. A purely intradural approach would have failed to address the extradural component of the aneurysm. Conversely, a purely extradural first approach, without initial exposure of the intradural ICA, would have been insufficient given the need for immediate distal control to manage the high risk of rupture while drilling the clinoid. The initial intradural exposure, followed by an intradural anterior clinoidectomy, and then a final dissection of the extradural dome, was the rational strategy for addressing the full extent of this transdural lesion. The advantage of a transcranial approach in this case was the ability to address the carotid terminus aneurysm at the same time. The morphology of the distal aneurysm, the patient’s young age, and the ability to treat both aneurysms simultaneously led the team to select a transcranial approach.

## Conclusions

This technical case description presents an instructive example of a paraclinoid ICA aneurysm with a paradoxical intradural and extradural anatomy. The patient’s aneurysm, with its intradural neck and partial extradural dome, highlights the limitations of preoperative imaging and bony landmark-based classification systems. Although a single case cannot establish generalizable diagnostic accuracy, this patient's anatomy, together with the existing literature, underscores the potential limitations of preoperative imaging and bony landmark classification systems, warranting a high degree of surgical preparedness. The successful obliteration of this complex lesion was made possible by a combined intradural and extradural strategy, which allowed for the establishment of secure distal control through an intradural anterior clinoidectomy before we proceeded with the extradural exposure and clipping. This case reinforces the fundamental neurosurgical principle that a thorough understanding of variable anatomy and the capacity to adapt the surgical technique to intraoperative findings are paramount for achieving a safe and favorable outcome.
